# Task-based functional neural correlates of social cognition across autism and schizophrenia spectrum disorders

**DOI:** 10.1186/s13229-024-00615-3

**Published:** 2024-09-04

**Authors:** Lindsay D. Oliver, Iska Moxon-Emre, Colin Hawco, Erin W. Dickie, Arla Dakli, Rachael E. Lyon, Peter Szatmari, John D. Haltigan, Anna Goldenberg, Ayesha G. Rashidi, Vinh Tan, Maria T. Secara, Pushpal Desarkar, George Foussias, Robert W. Buchanan, Anil K. Malhotra, Meng-Chuan Lai, Aristotle N. Voineskos, Stephanie H. Ameis

**Affiliations:** 1 Campbell Family Mental Health Research Institute, Centre for Addiction and Mental Health, Toronto, ON Canada; 2 Department of Psychiatry, Temerty Faculty of Medicine, University of Toronto, Toronto, ON Canada; 3 Institute of Medical Science, University of Toronto, Toronto, ON Canada; 4 Research Institute & Department of Psychiatry, The Hospital for Sick Children, Toronto, ON Canada; 5 Child and Youth Psychiatry Division, Centre for Addiction and Mental Health, Toronto, ON Canada; 6 Genetics & Genome Biology, The Hospital for Sick Children, Toronto, ON Canada; 7 Department of Computer Science, University of Toronto, Toronto, ON Canada; 8 Vector Institute, Toronto, ON Canada; 9 Temerty Centre for Therapeutic Brain Intervention, Centre for Addiction and Mental Health, Toronto, ON Canada; 10 Azrieli Adult Neurodevelopmental Centre, Centre for Addiction and Mental Health, Toronto, ON Canada; 11 Maryland Psychiatric Research Center, Department of Psychiatry, University of Maryland School of Medicine, Baltimore, MD USA; 12 Division of Psychiatry Research, Division of Northwell Health, The Zucker Hillside Hospital, Glen Oaks, NY USA; 13 Department of Psychiatry, The Donald and Barbara Zucker School of Medicine at Hofstra/Northwell, Hempstead, NY USA; 14 Center for Psychiatric Neuroscience, The Feinstein Institute for Medical Research, Manhasset, NY USA; 15 Department of Psychology, University of Toronto, Toronto, ON Canada; 16 Autism Research Centre, Department of Psychiatry, University of Cambridge, Cambridge, UK; 17 Department of Psychiatry, National Taiwan University Hospital and College of Medicine, Taipei, Taiwan

**Keywords:** Social cognition, Autism, Schizophrenia spectrum disorders, fMRI

## Abstract

**Background:**

Autism and schizophrenia spectrum disorders (SSDs) both feature atypical social cognition. Despite evidence for comparable group-level performance in lower-level emotion processing and higher-level mentalizing, limited research has examined the neural basis of social cognition across these conditions. Our goal was to compare the neural correlates of social cognition in autism, SSDs, and typically developing controls (TDCs).

**Methods:**

Data came from two harmonized studies in individuals diagnosed with autism or SSDs and TDCs (aged 16–35 years), including behavioral social cognitive metrics and two functional magnetic resonance imaging (fMRI) tasks: a social mirroring Imitate/Observe (ImObs) task and the Empathic Accuracy (EA) task. Group-level comparisons, and transdiagnostic analyses incorporating social cognitive performance, were run using FSL’s PALM for each task, covarying for age and sex (1000 permutations, thresholded at *p* < 0.05 FWE-corrected). Exploratory region of interest (ROI)-based analyses were also conducted.

**Results:**

ImObs and EA analyses included 164 and 174 participants, respectively (autism *N* = 56/59, SSD *N* = 50/56, TDC *N* = 58/59). EA and both lower- and higher-level social cognition scores differed across groups. While canonical social cognitive networks were activated, no significant whole-brain or ROI-based group-level differences in neural correlates for either task were detected. Transdiagnostically, neural activity during the EA task, but not the ImObs task, was associated with lower- and higher-level social cognitive performance.

**Limitations:**

Despite attempting to match our groups on age, sex, and race, significant group differences remained. Power to detect regional brain differences is also influenced by sample size and multiple comparisons in whole-brain analyses. Our findings may not generalize to autism and SSD individuals with co-occurring intellectual disabilities.

**Conclusions:**

The lack of whole-brain and ROI-based group-level differences identified and the dimensional EA brain-behavior relationship observed across our sample suggest that the EA task may be well-suited to target engagement in novel intervention testing. Our results also emphasize the potential utility of cross-condition approaches to better understand social cognition across autism and SSDs.

**Supplementary Information:**

The online version contains supplementary material available at 10.1186/s13229-024-00615-3.

## Background

Autism spectrum disorder (ASD; hereafter, referred to as autism) and schizophrenia spectrum disorders (SSDs) share social cognitive impacts that influence functioning and quality of life [1, 2]. There are currently no approved interventions nor established biomarkers to guide novel interventions for social cognitive impairments in either condition [3]. Although cross-condition research is increasing [4, 5], the extent to which neural correlates of social cognition are shared or distinct across the two conditions remains unclear.

Social cognition is subserved by partially dissociable neural circuits [6], including a ​​lower-level frontoparietal/mirror neuron simulation network for more basic emotion processing [7], and a higher-level cortical midline and lateral temporal mentalizing network for theory of mind [8, 9] (e.g., complex mental state inference). Via a meta-analysis, we recently found similar levels of behavioral lower- and higher-level social cognitive performance in SSDs and autism, though results were heterogeneous [4]. Prior autism-control research using functional magnetic resonance imaging (fMRI) tasks are suggestive of lower-level circuit alterations [10–12], with mixed evidence for higher-level circuit differences in autistic individuals versus controls [13, 14]. In people with SSDs, both lower-level [15] and higher-level [16–18] circuit differences versus controls have been shown. Few task-fMRI studies have compared autism and SSD samples directly, typically in smaller samples (Ns ~ 12-<50/group) using a single fMRI task. These studies have reported condition-specific neural signatures (e.g., increased activation [19] or increased connectivity [20, 21] in SSDs versus autism), but also a lack of group-level differences across autism and SSDs [21–24] in regions implicated in lower-level and higher-level social cognition.

Our primary objective was to examine common and unique differences in the neural correlates of lower-level and higher-level social cognition among individuals with autism or SSDs and typically developing controls (TDCs) using a group-wise whole-brain approach. We studied a unique transdiagnostic sample using harmonized multi-center neuroimaging and behavioral measures, including two different fMRI tasks: the Imitate/Observe (ImObs) task, which activates lower-level social cognitive regions [7, 25–27], and the Empathic Accuracy (EA) task to probe both lower-level simulation and higher-level mentalizing networks [28]. We hypothesized that the neural correlates of social cognition would differ between both autism and SSD compared to TDC groups, particularly in regions of the simulation and mentalizing networks. Our secondary objective was to examine the neural correlates of social cognition by stimuli valence (positive/negative), based on evidence of distinguishable brain responses to task emotional content [21, 29]. We hypothesized that neural correlates of social cognition would diverge across positive and negative stimuli, but that autism and SSD groups would differ from the TDC group, aligning with findings from the full tasks. Finally, we performed transdiagnostic whole-brain analyses to examine patterns of association between brain activity during the ImObs and EA tasks and lower- and higher-level social cognitive performance. We hypothesized that brain activation within circuitry implicated in lower- and higher-level social cognition (i.e., the simulation and mentalizing networks) would be dimensionally associated with lower- and higher-level social cognitive performance across groups. To complement the whole-brain analyses, exploratory post-hoc region of interest (ROI)-based analyses were conducted to examine effects of social cognitive performance and diagnostic group on regional brain activation across our sample.

## Methods

### Participants

Participants with a clinical diagnosis of autism and TDCs, aged 16–35 years, were recruited through our ongoing National Institute of Mental Health (NIMH) funded study (R01 MH114879, Social Processes Initiative in the Neurobiology of Autism-spectrum and Schizophrenia-spectrum Disorders, SPIN-ASD), from the Centre for Addiction and Mental Health (CAMH; Toronto, Canada) and local community agencies (August 2018-February 2023). Participants with a clinical SSD diagnosis and a TDC cohort, aged 18–59 years, were recruited through the completed partner harmonized NIMH-funded Research Domain Criteria study (1/3R01 MH102324, 2/3R0I MH102313, 3/3R01 MH102318): Social Processes Initiative in the Neurobiology of the Schizophrenia(s) (SPINS) [30–32]. SPINS participants were recruited from CAMH, Zucker Hillside Hospital (Glen Oaks, NY), and the Maryland Psychiatric Research Center (Baltimore, MD) (December 2014-December 2020). All SPIN-ASD assessments were harmonized to the SPINS study on clinical, cognitive, and imaging data collection, to examine the neurobiology of social cognition across autism, SSD, and TDC groups. From the total SPINS sample of 200 SSDs and 159 TDCs with usable EA fMRI data, a subset of SPINS participants, aged 16–35 and scanned on one of the three 3T Siemens Prisma scanners used across the SPINS sites, were included in the present study to optimize matching on age and scanner platform with SPIN-ASD. All included imaging data were collected on 3T Siemens Prisma scanners using matched scanning parameters and standardized operating procedures to minimize inter-site variance in task administration and data collection. Data were collected across three visits: screening and clinical assessments; MRI; neurocognitive and social cognitive assessments.

Participants with autism met DSM-5 criteria for ASD, confirmed based on a clinical interview conducted by a trained child and youth psychiatrist (SHA, M-CL, or other expert clinician from specialty autism assessment service), using the Autism Diagnostic Observation Schedule-2 (ADOS-2) [33]. Participants with SSDs met DSM-5 criteria for schizophrenia, schizophreniform disorder, delusional disorder, schizoaffective disorder, or psychotic disorder not otherwise specified, assessed using the Structured Clinical Interview for DSM (SCID-IV-TR). Included autism and SSDs participants were clinically stable, and had no changes in antipsychotic medication 30 days prior to study enrollment. TDC participants had no DSM-5 disorder, and had no first-degree relatives with a DSM-5 diagnosis of ASD, SSDs, or other psychotic disorders, or a bipolar or depressive disorder with psychotic features, confirmed using the SCID-IV-TR. Participants, regardless of diagnosis, were excluded if they: were non-English speakers, pregnant, had MRI contraindications (e.g., metal implants, pacemaker), IQ < 70 (based on WASI-II [34] or Wechsler Test of Adult Reading (WTAR) [35]), substance use/dependency disorder within the last 6 months or a positive baseline urine drug screen, prior psychosurgery, a history of head trauma resulting in unconsciousness for > 30 min, Type 1 diabetes mellitus, debilitating or unstable medical illness (e.g., cardiac, hepatic, renal or pulmonary disease, cancer), neurological diseases (e.g., Parkinson’s disease, epilepsy) or any central nervous system disorder. All autism, SSD, and TDC participants provided voluntary informed consent, and the SPIN-ASD and SPINS protocols were approved by the respective research ethics and institutional review boards across sites (CAMH Research Ethics Board, Northwell Health Human Research Protection Program, Institutional Review Board at the University of Maryland Baltimore).

### Social cognitive measures

Social cognition was assessed using EA task performance during fMRI, and the Reading the Mind in the Eyes Test (RMET), which involves identifying emotional and mental states based on pictures of the eye region only [36], the Penn Emotion Recognition Test (ER-40), a static facial emotion recognition task [37], and all subscales from the Awareness of Social Inference Test–Revised (TASIT), a task which probes both lower-level emotion processing and higher-level mentalizing via video vignettes [38], outside the scanner. Using this battery in the SPINS sample, we previously showed very good fit of a two-factor model including lower-level ‘simulation’ and higher-level ‘mentalizing’ factors across individuals with SSDs and TDCs [31]. In the current sample, scores from these measures were used to estimate lower-level ‘simulation’ (including ER-40, RMET, TASIT 3 Lies, and EA task scores) and higher-level ‘mentalizing’ (TASIT 2 Simple Sarcasm, TASIT 2 Paradoxical Sarcasm, and TASIT 3 Sarcasm) scores for each participant using multiple regression in the R package lavaan [39]. See Oliver et al., 2019 for details [31]. The EA task and out-of-scanner social cognitive tasks were selected based on findings from the Social Cognition Psychometric Evaluation (SCOPE) study [40] and the Social Cognition and Functioning in Schizophrenia project [41]. The ImObs and EA tasks are both established fMRI paradigms known to engage regions of the lower-level simulation network [7, 25–27], and both the lower- and higher-level social cognitive networks [28], respectively. The EA task is also considered to be a more naturalistic social cognitive task [42].

### Imaging

#### MRI data acquisition

MRI data from SPINS used in the present analyses were collected across three 3T Siemens Prisma scanners with multichannel head coils using harmonized scanning parameters, one of which was also used for MRI data collection in SPIN-ASD. Though SPINS also collected data on three other 3T MRI scanners, only data collected on Prisma scanners were included in our analyses to mitigate systematic study-related and scanner manufacturer/model measurement biases. Prior work has demonstrated the contribution of scanner manufacturer to measurement bias and site effects, in structural [43] and functional [44] MRI data. A lack of scanner-based differences in fMRI patterns has also been found across scanners from the SPINS study using traveling human phantom data [45]. See Table S1 for the number of scans by site, task, and diagnostic group. A T1-weighted anatomical scan (fast gradient sequence; TR = 2300 ms, TE = 3 ms, flip angle = 9*°*, field of view (FOV) = 230 mm, in plane resolution = 0.9 mm^2^, slice thickness = 0.9 mm), the EA task, and ImObs task were administered as part of the matched SPINS and SPIN-ASD multimodal MRI protocols [30]. Scans were checked by research staff, which included quantitative monitoring (e.g., mean framewise displacement) and qualitative monitoring (e.g., ringing or ghosting in scans).

##### ImObs task

The ImObs fMRI task [7, 10, 46] consists of separate counter-balanced Imitate and Observe runs (TR = 3000 ms, TE = 30 ms, flip angle = 77*°*, FOV = 192 mm, in plane resolution = 3 mm^2^, slice thickness = 3 mm). Participants viewed color photographs of 16 individuals (8 males/8 females) expressing five facial expressions (angry, fearful, sad, happy, or neutral), and intermittent fixation trials. During the imitate session, participants were instructed to imitate the expression shown on the faces in the photographs, and to only use their facial muscles in doing so to minimize motion. During the observe session, participants were instructed to observe the faces in the photographs without moving. Prior to the MRI, participants were carefully trained to perform the imitate task without head motion. In each session, there were 80 faces (16/expression) and 16 fixation trials that were presented in a pseudorandomized order. Each trial lasted approximately three seconds, with faces presented for two seconds, and an inter-stimulus interval jittered at 500–1500 ms. Performance was monitored via a camera positioned in the MRI, to ensure participants were performing the task correctly. ImObs fMRI data were excluded for participants that: (1) were missing data, (2) had a mean framewise displacement (FD) > 0.5 mm for either the imitate or observe runs, (3) failed imaging quality control (see Table S3).

##### EA task

The EA fMRI task [28, 41, 47] consists of three runs acquired using an echo-planar imaging sequence (TR = 2000 ms, TE = 30 ms, flip angle = 77°, FOV = 218 mm, in-plane resolution = 3.4 mm^2^, slice thickness = 4 mm). Participants watched 9 videos (~2.0–3.0 min each, three per run) of individuals detailing autobiographical events, and two interleaved control videos per run (40 s each). Using a button box, participants provided continuous ratings of how positive or negative they thought the individual felt on a 9-point scale (1 = extremely negative, 9 = extremely positive). An EA score was calculated for each participant by correlating their ratings over time with ratings provided by individuals in the videos, followed by Fisher r-to-z transformation [28]. As a behavioral control condition, participants provided continuous ratings corresponding to the lightness/darkness of a greyscale circle that changed shades (on a 9-point scale; 1 = extremely light, 9 = extremely dark) [30, 48]. A circle block score was calculated for each participant by correlating their ratings with standard ratings corresponding to the lightness/darkness of the circle, followed by Fisher r-to-z transformation. The control (circles) task was included to confirm that participants were engaged throughout the task. EA fMRI data were excluded for participants that: (1) were missing data for any of the three EA runs, (2) made no responses in any of the nine videos, (3) made only one (i.e., a single) button press across more than one of the nine videos, (4) had a mean circle block score < 0.2, (5) had a mean FD > 0.5 mm for any of the three EA runs, (6) failed imaging quality control (see Table S2).

#### MRI preprocessing

MRI preprocessing was performed using fMRIPrep 1.5.8 [49, 50], based on Nipype 1.4.1 [51]. Anatomical T1-weighted images were corrected for intensity non-uniformity and skull-stripped using ANTs 2.2.0 [52]. Brain tissue segmentation of cerebrospinal fluid, white-matter and gray-matter was performed using FSL 5.0.9 [53]. Brain surfaces were reconstructed using FreeSurfer 6.0.1 [54]. For each of the fMRI runs, fieldmap-less distortion correction was performed using ANTs [55]. Functional data was coregistered to the corresponding T1-weighted image using Freesurfer’s boundary-based registration with six degrees of freedom. Functional data underwent slice-timing correction and motion correction using MCFLIRT (FSL 5.0.9) [56].

The ciftify bids app (https://github.com/edickie/ciftify*)* [57] was then used to transform the functional data onto the cortical surface using a non-linear transform to the MNI152 template via FSL’s FNIRT. The ciftify_clean_img function from the ciftify toolbox was used to drop four TRs for each EA and ImObs scan, and to smooth the functional data using a 6 mm full width at half maximum Gaussian kernel.

### Statistical analyses

EA, simulation, and mentalizing scores were compared between autism, SSD, and TDC groups using Kruskal-Wallis tests due to non-normal distributions, followed by pairwise Dunn’s tests with false discovery rate (FDR) correction where applicable. These models were re-run after removing data points detected as outliers in sensitivity analyses (see Supplementary Methods).

Individual-level task activity was measured via general linear models (GLMs) using AFNI’s 3dDeconvolve module in nipype [58, 59]. Both ImObs and EA included event regressors using the standard hemodynamic response function (HRF), and noise regressors including the six head motion correction parameters, mean white matter signal, mean cerebral spinal fluid signal, and the square, derivative, and square of the derivative for each of these regressors (generated by fMRIPrep).

#### ImObs task

Event regressors for each expression and the fixation cross were modeled for each condition (imitate and observe). These regressors were fit to each voxel to model the stimulus-evoked response. Emotional faces were contrasted in the imitate versus the observe run. Contrasts were also generated for imitation versus observation of negative (sad, angry, fearful) and positive (happy) emotional faces separately.

#### EA task

Stimulus regressors were fit for the duration of the EA videos, circle videos, and for each button press. A parametric modulator regressor was also fit based on the EA score for each video, measuring brain activity associated with EA performance [16, 28, 30]. These regressors were fit to each voxel to model the stimulus-evoked response. A GLM was also run to model negative and positive videos separately, with the other regressors remaining the same.

Group-level task activity was assessed using 1000 permutations with Family-Wise Error (FWE)-corrected threshold-free cluster enhancement (TFCE: p_FWE_< 0.05), implemented in FSL’s Permutation Analysis of Linear Models (PALM) [60]. All PALM models included age and sex assigned at birth as covariates. For ImObs, beta maps from the imitate-observe contrast for emotional faces were examined. For EA, activity related to EA (parametric modulation) was examined. Group analyses were run separately for autism, SSD, and TDC groups, and contrasting autism-TDC, SSDs-TDC, and autism-SSDs. Analyses were run across all stimuli, and by valence. Across participants, separate PALM models were also run for ImObs and EA videos (not modulated by EA scores) including simulation and mentalizing scores as predictors to identify activity related to social cognitive performance.

To complement the transdiagnostic whole-brain findings, exploratory post-hoc ROI-based analyses were also conducted. ROIs were defined using meta-analytic association test maps generated by Neurosynth for the term ‘mentalizing’ (151 studies), and a topic-based map which we refer to as ‘simulation’ (96 studies), with top-loading terms including ‘mirror’, ‘system’, ‘neuron’, ‘observation’, and ‘mns’ (55 topics) [61, 62] (see Supplementary Methods). A topic-based map was used for simulation because this term is also used in other fields and terminology varies when referring to this construct, whereas mentalizing is more widely agreed upon in the literature. ROIs were selected based on implication in lower-level simulation from the 55 topic map (left and right inferior frontal gyrus [IFG] into premotor cortex [7]) and higher-level mentalizing from the mentalizing term map (left and right temporoparietal junction [TPJ] and left and right anterior superior temporal sulcus [STS] [8, 9]). Beta weights reflecting brain activity during the EA videos were extracted from these six Neurosynth-defined ROIs using ciftify [57].

For each ROI, exploratory regression models were run to estimate the effects of relevant social cognitive performance (i.e., either simulation or mentalizing scores) on ROI-based brain activity, with FDR correction applied. Simulation scores were used in models for the lower-level ROIs (left/right IFG), and mentalizing scores were used in models for the higher-level ROIs (left/right TPJ and STS) to limit the number of comparisons. To evaluate whether a model including diagnostic group information performed better than the transdiagnostic model including only social cognitive performance, linear models were also conducted for ROI-based brain activity including social cognitive performance (simulation or mentalizing), diagnostic group, and social cognitive performance x diagnostic group, with FDR correction. These models were compared to models including only social cognitive performance as a predictor using the Akaike Information Criteria (AIC). Additionally, potential scanner effects on brain activation patterns were examined using ANOVAs for each of the ROIs, with FDR correction. Age and sex assigned at birth were included as covariates in all models.

## Results

### Participants

A total of 226 participants with fMRI data collected on one of three 3T Siemens Prisma scanners used across SPINS/SPIN-ASD were submitted to quality control. Following quality control, matching was undertaken to optimize matching between SPIN-ASD and SPINS samples on age, sex, and race as implemented in the MatchIt package in R [63] (see Supplementary Methods and Tables S2-S3). Our sample consisted of 174 participants with usable EA task data (see Table 1 for demographic and clinical characteristics). From this sample, 164 participants had usable ImObs task-fMRI data (autism: *n* = 56, 20.9 [3.92] years, 22-female; SSD: *n* = 50, 24.7 [4.44] years, 17-female; TDC: *n* = 58, 25.8 [3.96] years, 32-female; Table S4). Autism, SSD, and TDC groups differed in age, race, ethnicity, and education (all *p* < 0.05).

**Table 1:** Participant demographic and clinical characteristics for empathic accuracy (EA) sample

	Sample with EA fMRI data				*P*-value
	Full sample (*n* = 174)	Aut (*n* = 59)	SSD (*n* = 56)	TDC (*n* = 59)	
**Age (years)**					**< 0.001**
Mean (SD)	24.1 (± 4.62)	21.2 (± 3.97)	25.3 (± 4.54)	25.8 (± 3.93)	
Median [Min, Max]	24.0 [16.0, 34.0]	21.0 [16.0, 33.0]	24.5 [18.0, 34.0]	26.0 [17.0, 34.0]	
**Sex**					0.07
Female	74 (43%)	23 (39%)	19 (34%)	32 (54%)	
Male	100 (57%)	36 (61%)	37 (66%)	27 (46%)	
**Handedness**					0.10
Right	144 (83%)	49 (83%)	47 (84%)	48 (81%)	
Left	19 (11%)	7 (12%)	2 (4%)	10 (17%)	
Mixed	1 (1%)	1 (2%)	0 (0%)	0 (0%)	
Missing	10 (5.7%)	2 (3.4%)	7 (12.5%)	1 (1.7%)	
**Race**					**< 0.001**
White	97 (56%)	44 (75%)	18 (32%)	35 (59%)	
Black or African American	27 (16%)	1 (2%)	18 (32%)	8 (14%)	
Asian	32 (18%)	4 (7%)	13 (23%)	15 (25%)	
More than one race	13 (7%)	6 (10%)	6 (11%)	1 (2%)	
Other	5 (3%)	4 (7%)	1 (2%)	0 (0%)	
**Ethnicity**					**0.02**
Hispanic or Latino	16 (9%)	9 (15%)	6 (11%)	1 (2%)	
Not Hispanic or Latino	158 (91%)	50 (85%)	50 (89%)	58 (98%)	
**Education (years)**					**< 0.001**
Mean (SD)	14.3 (± 2.47)	12.7 (± 2.13)	13.8 (± 1.76)	16.4 (± 1.86)	
Median [Min, Max]	14.0 [10.0, 20.0]	12.0 [10.0, 19.0]	13.0 [10.0, 18.0]	16.0 [11.0, 20.0]	
**Estimated IQ**					0.08
Mean (SD)	113 (± 12.6)	115 (± 13.8)	110 (± 12.3)	113 (± 11.2)	
Median [Min, Max]	115 [73.0, 145]	117 [73.0, 145]	113 [77.0, 127]	116 [79.0, 129]	
**BSFS Total**					**< 0.001**
Mean (SD)	147 (± 32.8)	125 (± 25.0)	137 (± 26.1)	178 (± 18.5)	
Median [Min, Max]	145 [54.0, 217]	127 [54.0, 180]	136 [75.0, 207]	178 [135, 217]	
**BPRS Total**					**0.03**
Mean (SD)	**-**	27.2 (± 4.9)	30.8 (± 8.29)	**-**	
Median [Min, Max]	**-**	27.0 [18.0, 40.0]	30.0 [19.0, 54.0]	**-**	
**ADOS-CSS (Aut Only)**					
Mean (SD)	**-**	6.39 (± 2.21)	**-**	**-**	
Median [Min, Max]	**-**	7.00 [1.00, 10.0]	**-**	**-**	
**SANS Total (SSD Only)**					
Mean (SD)	**-**	**-**	30.8 (± 8.29)	**-**	
Median [Min, Max]	**-**	**-**	30.0 [19.0, 54.0]	**-**	

Demographic and clinical characteristics for the full sample with usable empathic accuracy (EA) fMRI data. Age, education, and estimated IQ were compared across groups using non-parametric Kruskal-Wallis Rank Sum Tests given non-equal distributions. Sex was compared across groups using Chi-Square Test, whereas handedness, race, and ethnicity were compared using Fisher’s Exact Tests given cell values were < 5. Aut: autism; SSD: schizophrenia spectrum disorders; TDC: typically developing controls; BSFS: Birchwood Social Functioning Scale; BPRS: Brief Psychiatric Rating Scale; ADOS-CSS: Autism Diagnostic Observation Schedule - Calibrated Severity Scores; SANS: Scale for the Assessment of Negative Symptoms

### Social cognitive performance

**Fig. 1:**
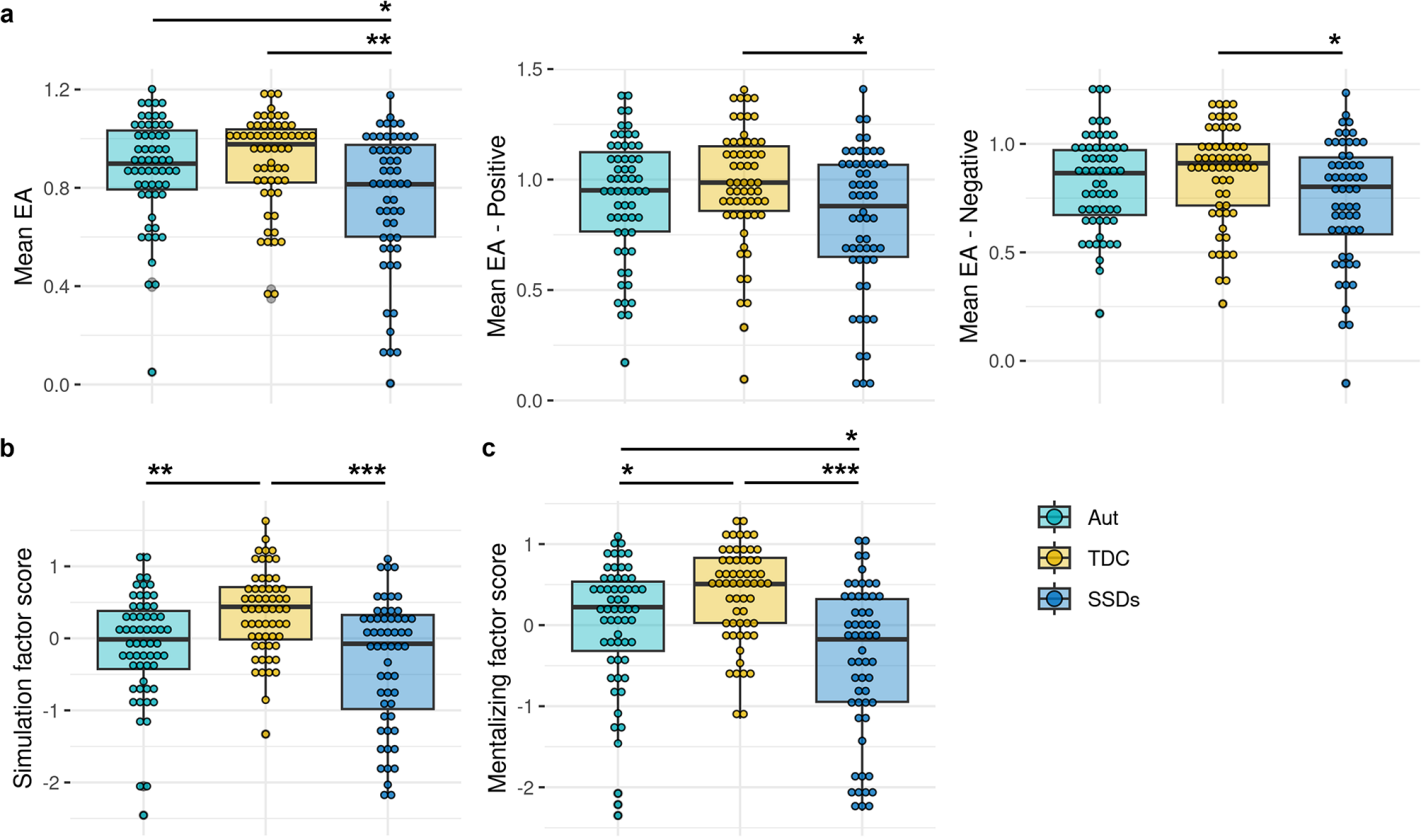
Group comparisons of social cognitive performance. (**a**) Empathic accuracy (EA) task performance from the full task, positive videos, and negative videos are shown, as well as (**b**) lower-level simulation scores and (**c**) higher-level mentalizing scores. Scores were compared across groups using non-parametric Kruskal-Wallis tests, followed by pairwise Dunn’s tests with false discovery rate correction. Aut: autism; SSDs: schizophrenia spectrum disorders; TDC: typically developing controls. *p < 0.05, **p < 0.01., ***p < 0.001

Autism, SSD, and TDC groups differed on full EA task scores (H(2) = 11.65, *p* = 0.003), EA positive videos (H(2) = 6.67, *p* = 0.04), and EA negative videos (H(2) = 6.90, *p* = 0.03; Fig. 1a). The SSD group had lower scores than the TDC (pFDR = 0.003) and autism (pFDR = 0.03) groups on the full EA task, and lower scores than the TDC group on EA positive (pFDR = 0.03) and EA negative (pFDR = 0.03) videos.

Simulation scores also differed across groups (H(2) = 20.67, *p* < 0.001), with both the SSD (pFDR < 0.001) and autism (pFDR = 0.001) groups scoring lower than TDCs (Fig. 1b). Groups differed on mentalizing scores (H(2) = 26.95, *p* < 0.001), where the SSD group scored lower than TDC (pFDR < 0.001) and autism (pFDR = 0.01), and the autism group scored lower than TDCs (pFDR = 0.01) (Fig. 1c; Table S5). Patterns of significant group differences in social cognitive performance remained the same in sensitivity analyses following the removal of detected outliers (see Supplementary Results).

### Group-level fMRI task activity

**Fig. 2:**
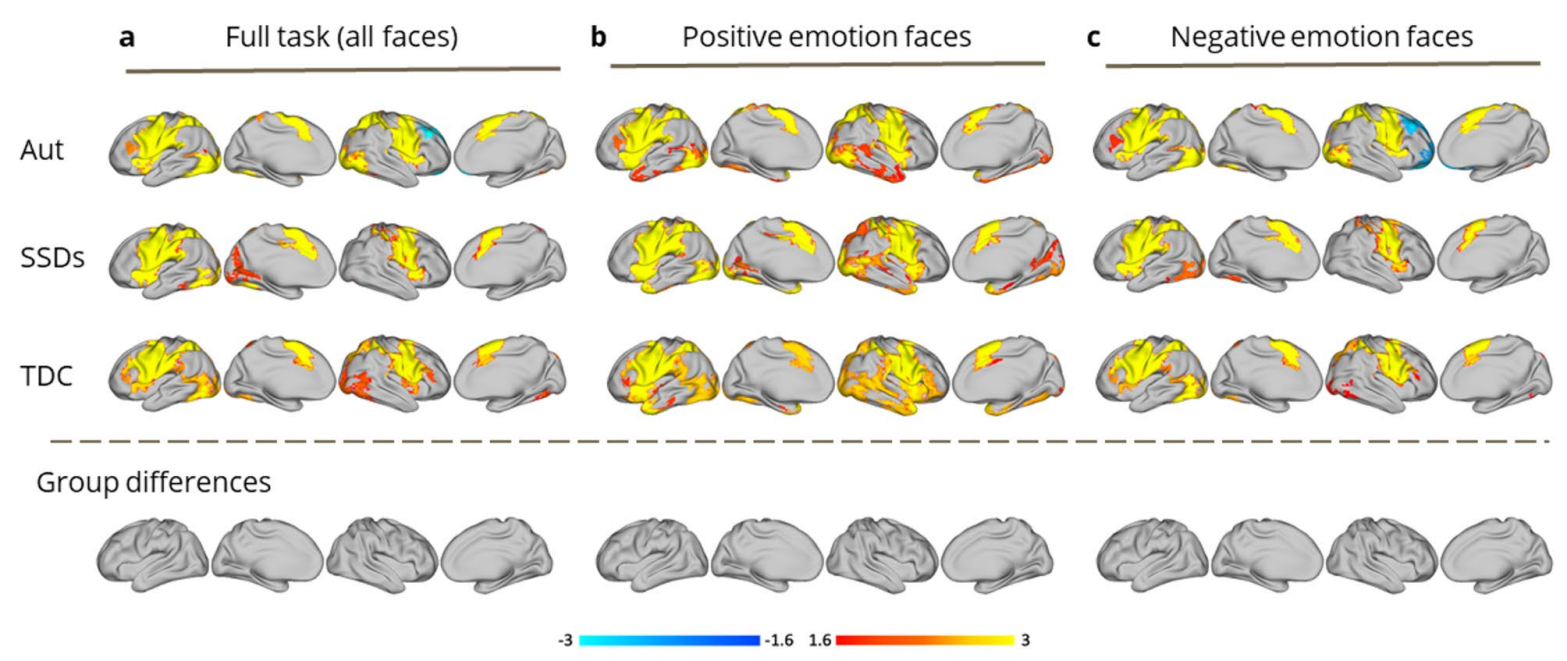
Group-level general linear model (GLM) analyses of Imitate/Observe (ImObs) task-fMRI data, conducted using FSL’s permutation analysis of linear models (PALM), for autism (Aut), schizophrenia spectrum disorders (SSDs), and typically developing control (TDC) groups. Main effects for each group are shown, according to analyses performed using: (**a**) all faces from the full ImObs task (*n* = 80); (**b**) positive emotion faces from the ImObs task (happy; *n* = 16); (**c**) negative emotion faces from the ImObs task (sad, angry, and fearful; *n* = 48). No significant group differences were detected

ImObs: All three groups demonstrated the expected pattern of activity during the ImObs task, including activation in the frontoparietal mirror neuron system (e.g., the IFG, premotor cortex, inferior parietal lobule, and posterior STS) [7, 10, 64] (Fig. 2). Results remained similar when trials containing positive or negative faces were considered separately. There were no significant group differences in activation for the full task, or for positive/negative faces (Fig. 2).

**Fig. 3:**
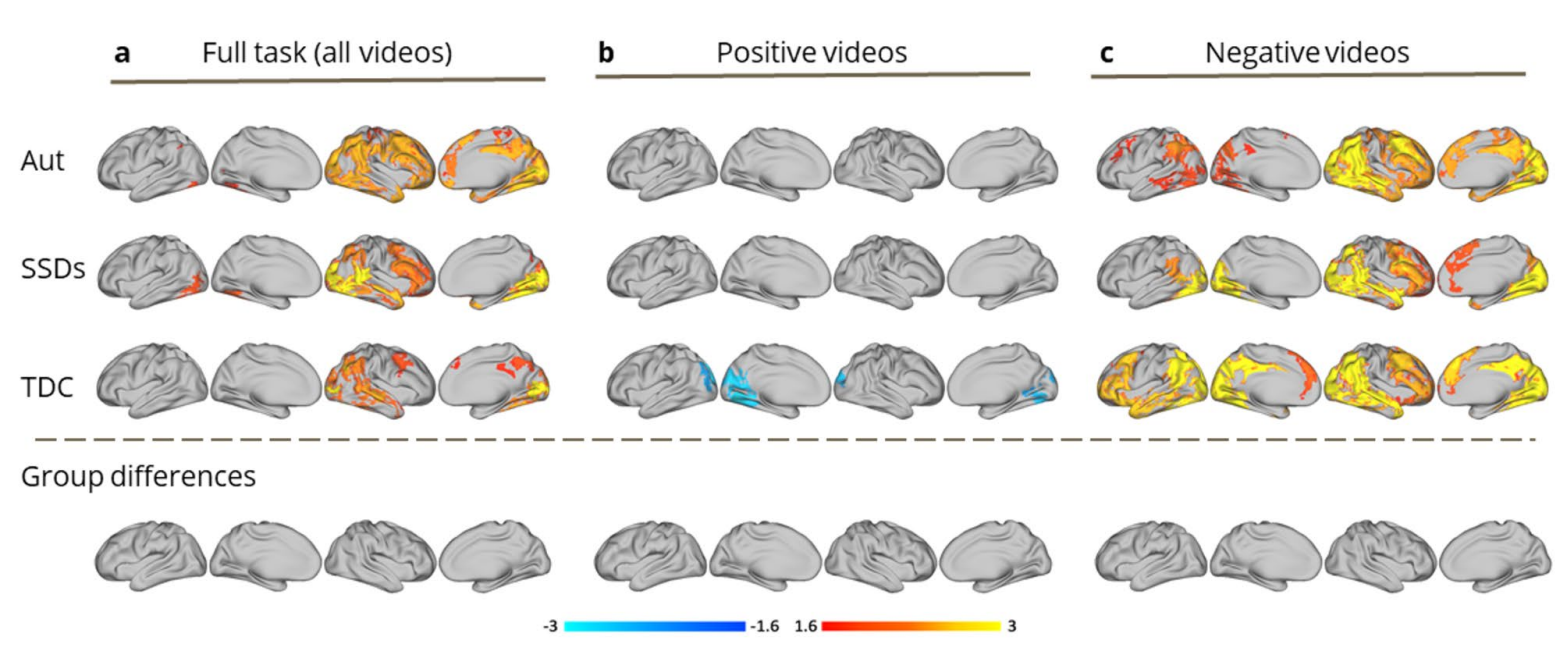
Group-level general linear model (GLM) analyses of empathic accuracy (EA) task-fMRI data, conducted using FSL’s permutation analysis of linear models (PALM), for autism (Aut), schizophrenia spectrum disorders (SSDs), and typically developing control (TDC) groups. Main effects for each group are shown, according to analyses performed using: (**a**) all videos from the EA task (*n* = 9); (**b**) positive videos from the EA task (*n* = 4); (**c**) negative videos from the EA task (*n* = 5). No significant group differences were detected

EA task: Across the full EA task, all groups demonstrated widespread activity in the right hemisphere that was positively related to EA performance, including in expected regions implicated in social cognition (e.g., temporal pole, STS, inferior parietal lobule, TPJ). During positive videos, no neural correlates of EA were significant in the autism and SSDs groups, though activity in visual regions were negatively related to EA in TDCs. During negative videos, all groups demonstrated widespread bilateral activity that was positively related to EA in brain regions implicated in social cognition. There were no significant group-level differences in neural correlates for EA for the full task or for positive/negative videos (Fig. 3).

### Transdiagnostic brain-behavior associations

**Fig. 4:**
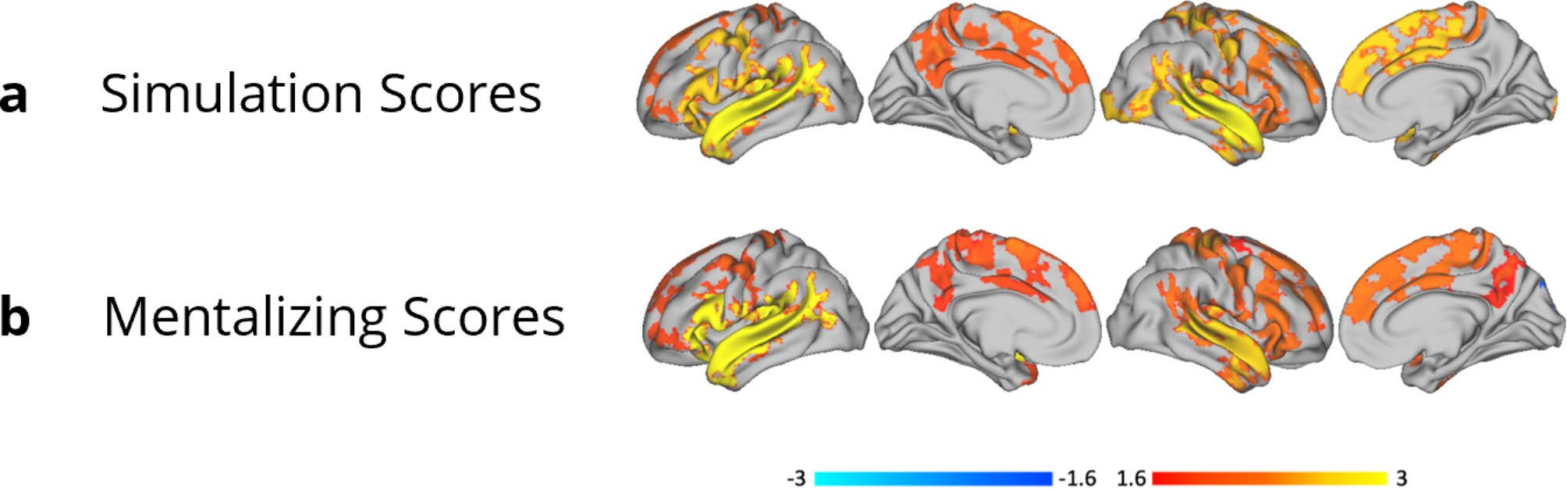
Transdiagnostic general linear model (GLM) analyses to examine relationships between empathic accuracy (EA) task-fMRI whole block data and social cognitive performance. Analyses were conducted using FSL’s permutation analysis of linear models (PALM) across diagnostic groups. Main effects shown here indicate regions where EA task activity (without parametric modulation) was related to (**a**) simulation and (**b**) mentalizing factor scores

Neither simulation nor mentalizing scores were significantly associated with brain activity during the ImObs task across participants. Both simulation and mentalizing scores were transdiagnostically positively related to widespread bilateral activation during the EA task in regions of the simulation and mentalizing networks, including the IFG, anterior insula, TPJ, and STS (Fig. 4).

### Exploratory region of interest (ROI)-based analyses

**Fig. 5:**
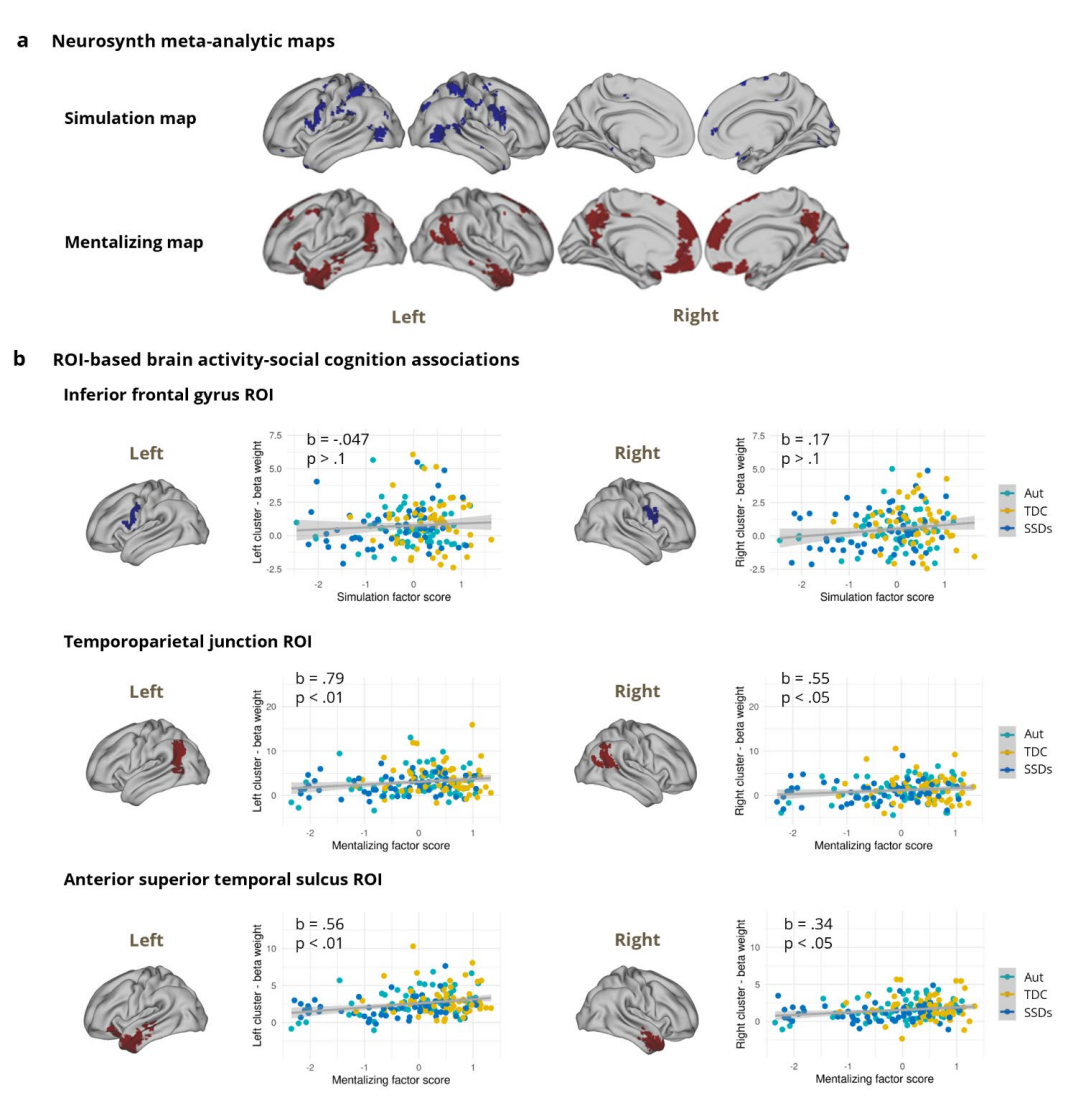
Exploratory associations between region of interest (ROI)-based brain activation and social cognitive performance scores. (**a**) Neurosynth meta-analytic maps used to define ROIs based on the term ‘mentalizing’ (red), and a topic-based map which we refer to as ‘simulation’, with top-loading terms including ‘mirror’, ‘system’, ‘neuron’, ‘observation’, and ‘mns’ (blue). (**b**) ROIs were selected from these clustered maps, including the left and right inferior frontal gyrus [IFG] into premotor cortex for lower-level simulation (blue) and the left and right temporoparietal junction [TPJ] and left and right anterior superior temporal sulcus [STS] for higher-level mentalizing (red). Transdiagnostic associations are shown between beta weights extracted from lower-level ROIs and simulation scores, and higher-level ROIs and mentalizing scores. Participant data points are colored by diagnostic group. Aut: autism; SSDs: schizophrenia spectrum disorders; TDC: typically developing controls

Exploratory post-hoc ROI-based regression analyses indicated that mentalizing scores were a significant predictor of brain activation in the left TPJ (b = 0.79, t(170) = 2.98, pFDR = 0.0099), right TPJ (b = 0.55, t(170) = 2.23, pFDR = 0.041), left STS (b = 0.56, t(170) = 3.85, pFDR = 0.001), and right STS (b = 0.34, t(170) = 2.72, pFDR = 0.014) across our sample, whereas simulation scores did not significantly predict activity in the left IFG ROI (b=-0.047, t(170)=-0.27, pFDR = 0.79) or right IFG (b = 0.17, t(170) = 1.01, pFDR = 0.37; Fig. 5). Exploratory ANOVAs examining the effects of social cognitive performance, diagnostic group, and social cognitive performance x diagnostic group on ROI-based brain activity revealed significant effects of mentalizing scores for the right TPJ, left STS, and right STS (all pFDR < 0.05), but not diagnostic group or social cognition x diagnostic group across ROIs (see Table S6 for details and Figure S1 for associations between regional brain activity and social cognition by diagnostic group). The models with social cognitive performance alone performed better for ROI-based brain activation compared to the models including social cognitive performance x diagnostic group for the left and right IFG and the right TPJ, whereas including mentalizing x group improved model performance for the left STS. Models with and without interaction terms included performed similarly for the left TPJ and right STS (see Supplementary Results for details). There were no significant differences by scanner in ROI-based activation (see Supplementary Results and Figure S2).

## Discussion

In the present study, we examined the neural correlates of social cognition across a large sample of research participants with a clinical diagnosis of autism or SSDs, and in TDC participants characterized using harmonized protocols including two fMRI tasks (the ImObs task and the EA task), and an out-of-scanner social cognitive battery. We did not detect any group-level differences among autistic participants, people with SSDs, or TDCs in activity during the ImObs task, nor in activity modulated by EA task performance using whole-brain analyses or within select ROIs. Neural activity across simulation and mentalizing network regions during the EA task, but not the ImObs task, was positively associated with both lower- and higher-level social cognitive performance across our transdiagnostic sample.

The first important contribution to the field of the present work is the direct comparison of autistic individuals and those with SSDs using two well-established social brain tasks (ImObs/EA). While the expected pattern of activity was detected across groups during ImObs, including frontoparietal mirror neuron circuit activation [7], the lack of group differences detected in neural engagement (across the full task and valence-specific analyses) contrast with prior positive autism-TDC studies. A meta-analysis of several of those studies (albeit < 10 total) using action observation and imitation fMRI tasks, found increased simulation network activation in autism, though autism-TDC group differences did not survive rigorous thresholding [12]. A cross-condition study found no differential activation between autism and SSD groups during performance of an implicit facial affect recognition task; though, diminished activation in face processing regions in autism and SSDs versus TDCs was reported [24]. Our results, in a relatively large transdiagnostic sample, align with a study reporting no significant differences between SSDs and controls in activation during facial expression imitation versus observation [65].

We also detected no group-level differences in neural activity modulated by EA performance, either for the full task or analyzed by positive/negative valence. Across the full EA task, we observed widespread activity in brain regions implicated in lower- and higher-level social cognition within the right hemisphere related to EA performance across groups. Our valence-specific findings help explain this laterality, as the inclusion of positive EA videos during the full task may have masked the bilaterality of activation present in response to the negative videos. In general, negative stimuli are thought to be more complex, requiring greater attention and cognitive effort than positive stimuli [66]. Prior social cognitive fMRI research in relevant samples have reported inconsistent results. A small study found activity in left precuneus, middle frontal gyrus, and bilateral thalamus was more correlated with EA task performance in a TDC versus SSD group [16]. Opposing activation and functional connectivity patterns in autism versus SSDs, within mentalizing regions, was reported during a higher-level intention understanding task [20]. Others have reported reduced modulation of mentalizing regions during a mentalizing task [22] and differences in dynamic connectivity in both autism and SSD groups versus TDCs while performing a task similar to EA [21]. Inconsistencies in these results are likely contributed to by inclusion of small heterogeneous samples (~ 15–40/group), as well as differences in the methods and social cognitive tasks used.

Although individuals diagnosed with autism or SSDs often feature reduced social cognitive performance compared to controls, considerable heterogeneity in performance levels has been documented among individuals diagnosed with either condition [67]. The present lack of diagnosis-specific differences in whole-brain activation patterns may result from the degree of variability inherent within and across diagnostic groups and TDCs, rather than evidence that differences in social cognitive task-based brain activation, or social cognitive performance, do not exist between individuals with autism, SSDs, and controls, or subgroups of individuals. Prior work from our group suggests that individuals with autism and SSDs show greater individual variability in task-based fMRI brain activity [68, 69], which may obscure group-based statistical differences. Despite no significant group-based neural differences being detected at the whole-brain or ROI level in the current study, lower simulation and mentalizing scores were common to SSD and autism groups versus TDCs in our sample. Consistently, prior research has demonstrated the presence of meaningful differences in social cognition between individuals with SSDs or autism compared to typically developing individuals, though prior work also indicates that social cognitive performance can range from normative to very low across individuals diagnosed with either condition [67]. Given this heterogeneity, our brain-behavior results emphasize the need to incorporate other approaches alongside or in addition to conventional diagnostic group-based comparisons in future cross-condition social cognition research.

Both simulation and mentalizing scores showed a positive transdiagnostic association with activation in social cognitive regions while viewing EA videos in whole-brain analyses, suggesting that social cognitive performance tracks with brain activity during social processing across autism, SSDs, and TDCs to some degree and that dimensional analyses are useful to examine and parse these relationships. This aligns with previous work from our group demonstrating an association between functional connectivity during the EA task and social cognitive performance across SSDs and TDCs [48]. It is, however, important to note that our post-hoc analyses (comparing performance of models with or without a social cognition by diagnosis interaction term) indicated that models with social cognition alone outperformed those including diagnostic group in some, but not all, of the ROIs interrogated. This suggests that diagnostic group can provide additional useful information when examining brain activation during social processing across autism or SSDs groups and typically developing samples. Moreover, social cognitive performance and diagnosis were conflated in the current sample, as TDCs tend to score higher than participants in either clinical group. While a transdiagnostic approach may help distinguish the most impacted individuals across SSDs and autism groups from those performing more similarly to TDCs, biological mechanisms underlying social cognitive impacts may differ in different subgroups of participants, or from mechanisms underlying normative social cognitive performance and/or in those with/without clinical diagnoses, underlining the importance of continued efforts to understand the impact of diagnostic status [70, 71]. Further, activation during the ImObs task was not significantly associated with simulation or mentalizing scores across groups. Prior work using the ImObs data across SSDs, TDCs, and bipolar disorder identified clusters of participants (unrelated to diagnosis), with distinct neural activation patterns during this task, which also featured differences in social cognitive performance [64]. Taken together, our results support the incorporation in future cross-disorder research of dimensional brain-behavior analyses to understand circuits that underpin social cognitive performance across autism and SSDs and/or data-driven clustering to generate more homogeneous subgroups of participants, consistent with a focus in psychiatry towards disentangling heterogeneity [72–74], including both the RDoC framework and EU PRISM project [71, 75].

### Limitations

Despite attempting to match our groups on age, sex, and race, significant group differences remained, prompting our inclusion of age as a covariate in PALM analyses. Smaller numbers of females across our autism and SSD groups limit the power to test for potential sex effects across groups, and gender-related constructs were not measured across our sample [76]. While TFCE is a powerful approach for whole-brain analyses [60, 77], sample size limitations paired with examination of multiple dependent variables and the need to correct for multiple comparisons reduces statistical power [78]. Such factors may have limited the ability to detect diagnostic group-based differences in the current study. Though group-based differences were not evident in our ROI-based analyses, these were limited to six ROIs to reduce the number of comparisons. Therefore, the absence of evidence in our whole-brain analyses of any group-based differences in the present sample cannot be interpreted as evidence of the absence of any true differences between autism, SSDs, and TDC groups. Further, our findings may not generalize to autism and SSD individuals with co-occurring intellectual disability, given WASI-II/WTAR scores estimating IQ for our autism and SSD samples were within the average range. We also did not account for medication, though the lack of fMRI group-based differences suggests that this did not drive our results. Though we saw a lack of whole-brain group-based differences in activation during two observational social cognitive fMRI tasks, results may differ based on task sensitivity and ecological validity, and social interaction tasks in particular may provide additional insights into the neurobiological substrates of everyday social interactions [79]. While our study was not designed to test the conceptualization that autism and SSDs are diametrically opposed [20, 80], our results do not provide support for this hypothesis. While a unique strength of our study is the use of shared assessments, including clinical scales, future work needs to examine the influence of co-occurring mental health conditions on brain-behavior associations found across autism and SSDs [81].

## Conclusions

The present study did not identify whole-brain group-level differences in the functional neural correlates of social cognition across two fMRI tasks in autism, SSDs, and TDCs. Given the ability of the EA task to detect dimensional brain-behavior relationships that cut across two major diagnostic groups with relevance to social cognitive performance, this task may be particularly well-suited for target engagement in novel cross-condition biomarker testing. Further cross-condition work is needed based on our results, prior findings, and the hypothesized importance of social cognition as a driver of functional impact across autism and SSDs [82, 83]. The use of data-driven approaches to identify participants with more homogeneous neurobiological and clinical patterns may yield opportunities for prognostication and stratification into clinical trials.

## Electronic supplementary material

Below is the link to the electronic supplementary material.

Supplementary Material 1

## Data Availability

Code used for these analyses is available at https://github.com/loliver4/EA_ImObs_SPINS_SPASD. SPINS (ID #2098) and SPIN-ASD (ID #2923) data are currently available via the National Institute of Mental Health Data Archive. Researchers can submit a Data Access Request to request access to record-level human subject data for research purposes at https://nda.nih.gov/.

## Abbreviations

SSDs: Schizophrenia spectrum disorders
TDCs: Typically developing controls
fMRI: Functional magnetic resonance imaging
ImObs: Imitate/Observe
EA: Empathic Accuracy
NIMH: National Institute of Mental Health
SPIN-ASD: Social Processes Initiative in the Neurobiology of Autism-spectrum and Schizophrenia-spectrum Disorders
CAMH: Centre for Addiction and Mental Health
SPINS: Social Processes Initiative in the Neurobiology of the Schizophrenia(s)
WTAR: Wechsler Test of Adult Reading
RMET: Reading the Mind in the Eyes Test
ER-40: Penn Emotion Recognition Test
TASIT: The Awareness of Social Inference Test-Revised
FOV: Field of view
FD: Framewise displacement
FDR: False discovery rate
GLM: General linear model
HRF: Hemodynamic response function
FWE: Family-Wise Error
PALM: Permutation Analysis of Linear Models
ROIs: Regions of interest
IFG: Inferior frontal gyrus
TPJ: Temporoparietal junction
STS: Superior temporal sulcus
