# Supplementary material for: Task-based functional neural correlates of social cognition across autism and schizophrenia spectrum disorders
Source: Mol Autism. 2024 Sep 4;15:37. doi: 10.1186/s13229-024-00615-3 (PMC11385649; doi:10.1186/s13229-024-00615-3)
Supplement: Supplementary file 1 — Supplementary Material 1 [file 13229_2024_615_MOESM1_ESM.docx]

**Supplementary Material**

**Methods**

**Table S1.** Number of scans by site, task, and diagnostic group

**Table S2.** Empathic Accuracy (EA) sample quality control

**Table S3.** Imitate/Observe (ImObs) sample quality control

**Results**

**Table S4.** Participant demographic and clinical characteristics for Imitate/Observe (ImObs) sample

**Table S5.** Social cognitive performance

**Table S6.** Effects of social cognitive performance, diagnostic group, and social cognitive performance x diagnostic group on ROI-based brain activity

**Figure S1.** Associations between region of interest (ROI)-based brain activation and social cognitive performance scores by diagnostic group

**Figure S2.** Region of interest (ROI)-based brain activity by scanner

**References**

**Methods**

***Imaging***

**Table S1: Number of scans by site, task, and diagnostic group**


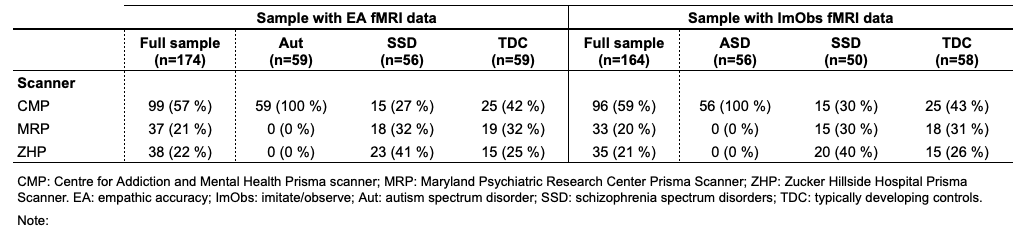


***Quality control (QC) and matching***

Prior to QC and matching, our sample consisted of 226 participants with fMRI data collected on Prisma scanners. Following QC, there were 190 participants available for matching (autism n=59; SSD n=62; TDC N=69). We matched the 59 autism participants with usable EA task fMRI data to SSDs (from the SPINS sample) and TDCs (from the combined SPINS and SPIN-ASD samples) with usable EA task fMRI data, resulting in a final sample of 59 Autism, 56 SSD, and 59 TDC. Groups were matched on age, sex, and race, as implemented in the MatchIt package in R [[1]](https://paperpile.com/c/zcPQRW/3qayC)*.* For full QC details, see Table S2.

**Table S2: Empathic Accuracy (EA) sample quality control**


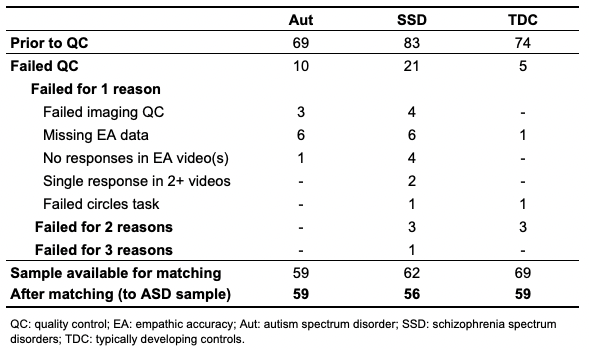


From our sample of 174 participants with usable EA task fMRI data, usable ImObs data were available from 164 participants, including 56 Autism, 50 SSD, and 58 TDC (see Table S3). We note that our sample was initially selected based on participants with usable EA data, given we expected fewer participants to pass QC criteria for the ImObs tasks (as it requires facial movement during “imitation” blocks). This approach was taken to avoid excluding individuals with usable EA data who did not have usable ImObs data.

**Table S3: Imitate/Observe (ImObs) sample quality control**

***
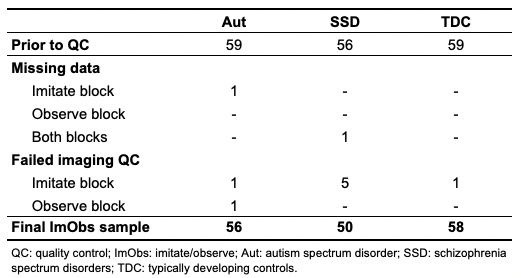
***

***Social cognitive performance sensitivity analyses***

Outliers were detected as data points more than 3 standard deviations from the mean across groups for each metric of social cognitive performance. The non-parametric group-based analyses were re-run after excluding these data points, including Kruskal-Wallis tests, followed by pairwise Dunn’s tests with false discovery rate (FDR) correction where applicable.

***Neurosynth-defined regions of interest (ROIs) for exploratory post-hoc analyses***

ROIs were defined using meta-analytic association test maps generated by Neurosynth for the term ‘mentalizing’ (151 studies), and a topic-based map which we refer to as ‘simulation’ (96 studies), with top-loading terms including ‘mirror’, ‘system’, ‘neuron’, ‘observation’, and ‘mns’ (55 topics) [[2,3]](https://paperpile.com/c/WcVrFf/AnVB+68Af). Neurosynth term-based association test maps depict brain regions that are preferentially related to a term based on study abstracts, whereas topic-based association maps display brain regions that are associated with a topic based on applying a topic modeling approach to article abstracts or text [[2,3]](https://paperpile.com/c/WcVrFf/AnVB+68Af). These meta-analytic maps were clustered using connectome workbench [[4]](https://paperpile.com/c/WcVrFf/jQqJ) and projected to the cortical surface using ciftify [[5]](https://paperpile.com/c/WcVrFf/lr0n).

**Results**

**Table S4: Participant demographic and clinical characteristics for Imitate/Observe (ImObs) sample**

|  | **Sample with ImObs fMRI data** | | | |  |
| --- | --- | --- | --- | --- | --- |
|  | **Full sample**  **(n=164)** | **Aut**  **(n=56)** | **SSD**  **(n=50)** | **TDC**  **(n=58)** | **P-value** |
| **Age (years)** |  |  |  |  | **<0.001** |
| Mean (SD) | 23.8 (± 4.59) | 20.9 (± 3.92) | 24.7 (± 4.44) | 25.8 (± 3.96) |  |
| Median [Min, Max] | 23.0 [16.0, 34.0] | 20.0 [16.0, 33.0] | 24.0 [18.0, 34.0] | 26.0 [17.0, 34.0] |  |
| **Sex** |  |  |  |  | 0.065 |
| Female | 71 (43 %) | 22 (39 %) | 17 (34 %) | 32 (55 %) |  |
| Male | 93 (57 %) | 34 (61 %) | 33 (66 %) | 26 (45 %) |  |
| **Handedness** |  |  |  |  | 0.22 |
| Right | 135 (82 %) | 46 (82 %) | 41 (82 %) | 48 (83 %) |  |
| Left | 18 (11 %) | 7 (12 %) | 2 (4 %) | 9 (16 %) |  |
| Mixed | 1 (1 %) | 1 (2 %) | 0 (0 %) | 0 (0 %) |  |
| Missing | 10 (6.1%) | 2 (3.6%) | 7 (14.0%) | 1 (1.7%) |  |
| **Race** |  |  |  |  | **<0.001** |
| White | 93 (57 %) | 42 (75 %) | 17 (34 %) | 34 (59 %) |  |
| Black or African American | 24 (15 %) | 0 (0 %) | 16 (32 %) | 8 (14 %) |  |
| Asian | 30 (18 %) | 4 (7 %) | 11 (22 %) | 15 (26 %) |  |
| More than one race | 12 (7 %) | 6 (11 %) | 5 (10 %) | 1 (2 %) |  |
| Other | 5 (3 %) | 4 (7 %) | 1 (2 %) | 0 (0 %) |  |
| **Ethnicity** |  |  |  |  | **0.017** |
| Hispanic or Latino | 15 (9 %) | 9 (16 %) | 5 (10 %) | 1 (2 %) |  |
| Not Hispanic or Latino | 149 (91 %) | 47 (84 %) | 45 (90 %) | 57 (98 %) |  |
| **Education (years)** |  |  |  |  | **<0.001** |
| Mean (SD) | 14.3 (± 2.52) | 12.6 (± 2.10) | 13.7 (± 1.74) | 16.4 (± 1.87) |  |
| Median [Min, Max] | 14.0 [10.0, 20.0] | 12.0 [10.0, 19.0] | 13.0 [10.0, 18.0] | 16.0 [11.0, 20.0] |  |
| **Estimated IQ** |  |  |  |  | 0.055 |
| Mean (SD) | 113 (± 12.7) | 115 (± 14.1) | 109 (± 12.1) | 113 (± 11.2) |  |
| Median [Min, Max] | 115 [73.0, 145] | 117 [73.0, 145] | 113 [77.0, 127] | 115 [79.0, 129] |  |
| **BSFS Total** |  |  |  |  | **<0.001** |
| Mean (SD) | 147 (± 33.1) | 125 (± 25.7) | 136 (± 25.1) | 178 (± 18.7) |  |
| Median [Min, Max] | 146 [54.0, 217] | 128 [54.0, 180] | 136 [75.0, 190] | 178 [135, 217] |  |
| **BPRS Total** |  |  |  |  | **0.027** |
| Mean (SD) | **-** | 27.1 (± 4.96) | 30.8 (± 8.21) | **-** |  |
| Median [Min, Max] | **-** | 27.0 [18.0, 40.0] | 30.0 [20.0, 54.0] | **-** |  |
| **ADOS-CSS (Aut Only)** |  |  |  |  |  |
| Mean (SD) | **-** | 6.14 (± 2.17) | **-** | **-** |  |
| Median [Min, Max] | **-** | 7.00 [1.00, 10.0] | **-** | **-** |  |
| **SANS Total (SSDs Only)** |  |  |  |  |  |
| Mean (SD) | **-** | **-** | 24.7 (± 12.9) | **-** |  |
| Median [Min, Max] | **-** | **-** | 24.0 [1.00, 54.0] | **-** |  |
| Demographic and clinical characteristics of the sample with usable imitate/observe (ImObs) fMRI data. Age, education, and estimated IQ were compared across groups using non-parametric Kruskal-Wallis Rank Sum Tests given non-equal distributions. Sex was compared across groups using Chi-Square Test, whereas handedness, race, and ethnicity were compared using Fisher's Exact Tests (cell values were <5). Aut: autism; SSD: schizophrenia spectrum disorders; TDC: typically developing controls; BSFS: Birchwood Social Functioning Scale; BPRS: Brief Psychiatric Rating Scale; ADOS-CSS: Autism Diagnostic Observation Schedule - Calibrated Severity Scores; SANS: Scale for the Assessment of Negative Symptoms. | | | | | |

**Table S5: Social cognitive performance**

**
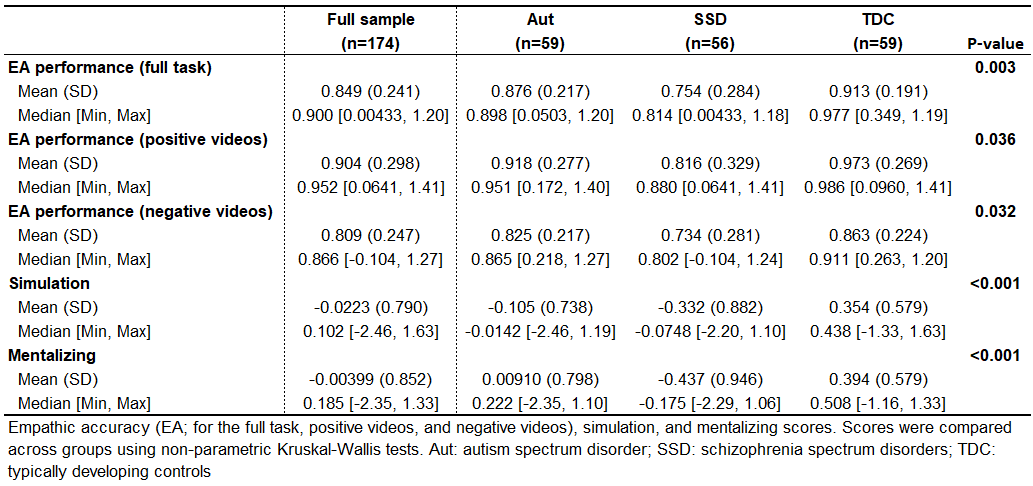
**

***Social cognitive performance sensitivity analyses***

After removing 3 outliers for mean EA (1 from the autism group, 2 from the SSD group) and 1 outlier for negative EA (from the SSD group), autism, SSD, and TDC groups still differed on full EA task scores (H(2)=10.13, p=0.006) and EA negative videos (H(2)=6.17, p=0.046). The SSD group still had lower scores than the TDC (pFDR=0.006) and autism (pFDR=0.03) groups on the full EA task, and lower scores than the TDC group on EA negative videos (pFDR=0.04). After removing 1 outlier from the autism group, simulation scores still differed across groups (H(2)=20.46, p<0.001), with both the SSD (pFDR<0.001) and autism (pFDR=0.001) groups scoring lower than the TDC group.

**Table S6: Effects of social cognitive performance, diagnostic group, and social cognitive performance x diagnostic group on ROI-based brain activity**

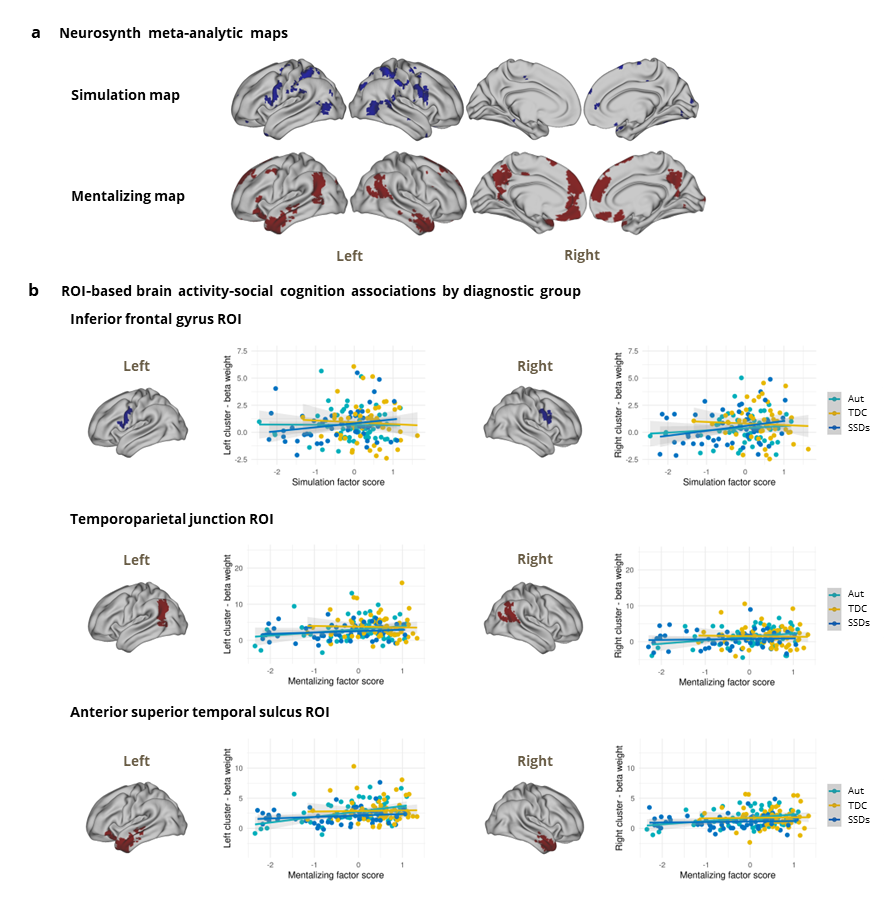


**Figure S1.** Exploratory associations between region of interest (ROI)-based brain activation and social cognitive performance scores by diagnostic group. (**a**) Neurosynth meta-analytic maps used to define ROIs based on the term ‘mentalizing’ (red), and a topic-based map which we refer to as ‘simulation’, with top-loading terms including ‘mirror’, ‘system’, ‘neuron’, ‘observation’, and ‘mns’ (blue). (**b**) ROIs were selected from these clustered maps, including the left and right inferior frontal gyrus [IFG] into premotor cortex for lower-level simulation (blue) and the left and right temporoparietal junction [TPJ] and left and right anterior superior temporal sulcus [STS] for higher-level mentalizing (red). Associations are shown between beta weights extracted from lower-level ROIs and simulation scores, and higher-level ROIs and mentalizing scores by diagnostic group. Participant data points are colored by diagnostic group. Aut: autism; SSDs: schizophrenia spectrum disorders; TDC: typically developing controls.

***Model Comparisons***

An AIC difference (ΔAIC) less than or equal to 2 indicates that both models are largely similar, whereas models with a difference of 4 or greater tend to show less support for the comparison model relative to the other, where lower AIC indicates better fit [[6]](https://paperpile.com/c/WcVrFf/4DNV). Some of the models with simulation or mentalizing performance alone (scog) performed better for ROI-based brain activation compared to the models including social cognitive performance x diagnostic group (scogXdx) according to AIC (i.e., lower AIC in more parsimonious model): the left IFG (scog AIC = 707, scogXdx AIC = 711, ΔAIC = 4) and right IFG (scog AIC = 683, scogXdx AIC = 689, ΔAIC = 6) with AIC differences >= 4, and to a lesser degree for the right TPJ (scog AIC = 856, scogXdx AIC = 859, ΔAIC = 3). However, the model including mentalizing performance x diagnostic group performed better than the model with mentalizing alone for the left STS (scog AIC = 669, scogXdx AIC = 665, ΔAIC = 4), and the models were similar to each other for the left TPJ (scog AIC = 877, scogXdx AIC = 879, ΔAIC = 2) and the right STS (scog AIC = 613, scogXdx AIC = 613, ΔAIC = 0) with AIC differences <=2.

***ROI-based brain activity by scanner***

There were no significant differences in brain activity by scanner (Figure S2) in left IFG (F(2,171)=0.19, pFDR=0.9), right IFG (F(2,171)=0.25, pFDR=0.9), left TPJ (F(2,171)=0.76, pFDR=0.9), right TPJ (F(2,171)=2.48, pFDR=0.5), left STS (F(2,171)=0.11, pFDR=0.9), or right STS (F(2,171)=0.63, pFDR=0.9).


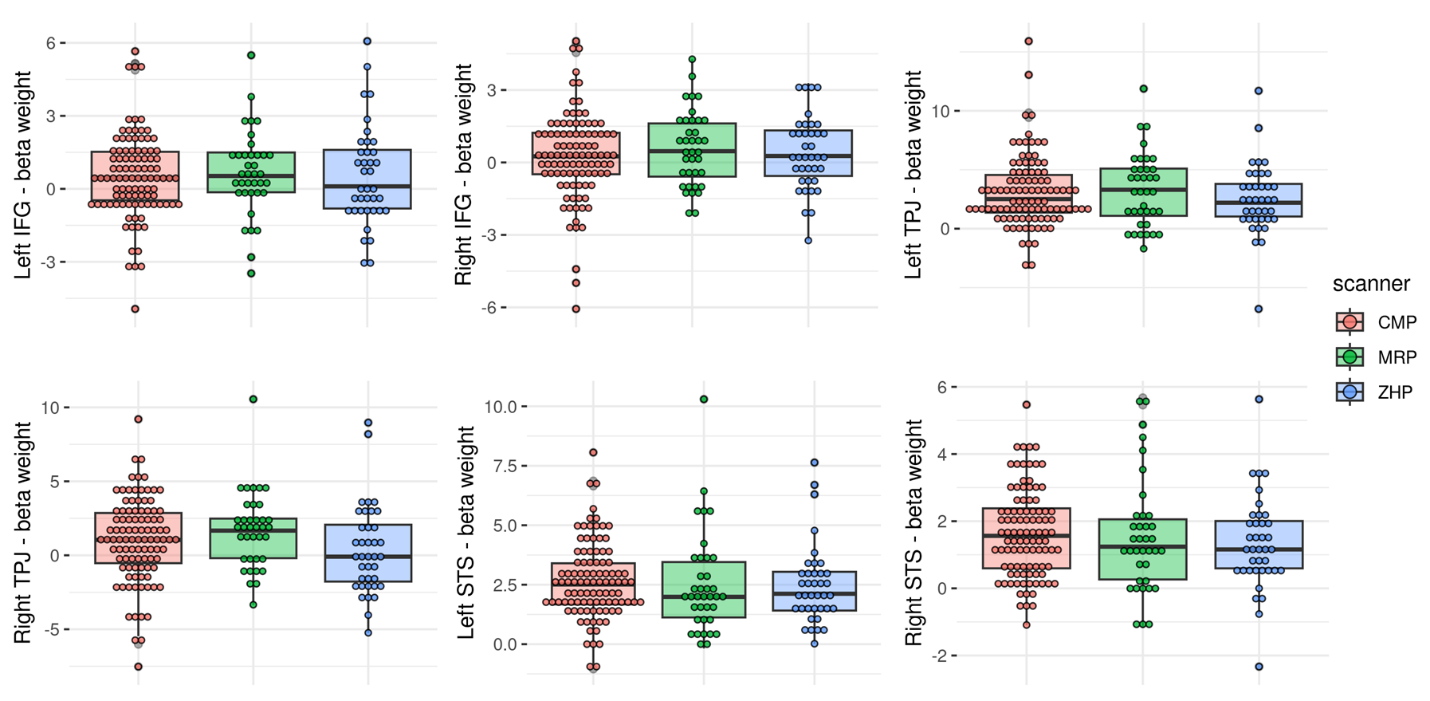


**Figure S2.** Region of interest (ROI)-based brain activity by scanner. Beta weights extracted during the empathic accuracy (EA) videos were compared based on the scanner used for data collection for each of the left and right inferior frontal gyrus (IFG), left and right temporoparietal junction (TPJ), and left and right anterior superior temporal sulcus (STS). Participant data points are colored by scanner. CMP: Centre for Addiction and Mental Health Prisma scanner; MRP: Maryland Psychiatric Research Center Prisma scanner; ZHP: Zucker Hillside Hospital Prisma scanner.

**References**

[1. Ho DE, Imai K, King G, Stuart EA. MatchIt: Nonparametric Preprocessing for Parametric Causal Inference. J Stat Softw [Internet]. 2011 [cited 2022 Dec 28];42. Available from:](http://paperpile.com/b/WcVrFf/fx1LO) <https://dash.harvard.edu/handle/1/11130519>

[2. Yarkoni T, Poldrack RA, Nichols TE, Van Essen DC, Wager TD. Large-scale automated synthesis of human functional neuroimaging data. Nat Methods. 2011;8:665–70.](http://paperpile.com/b/WcVrFf/AnVB)

[3. Poldrack RA, Mumford JA, Schonberg T, Kalar D, Barman B, Yarkoni T. Discovering relations between mind, brain, and mental disorders using topic mapping. PLoS Comput Biol. 2012;8:e1002707.](http://paperpile.com/b/WcVrFf/68Af)

[4. Marcus DS, Harms MP, Snyder AZ, Jenkinson M, Wilson JA, Glasser MF, et al. Human Connectome Project informatics: quality control, database services, and data visualization. Neuroimage. 2013;80:202–19.](http://paperpile.com/b/WcVrFf/jQqJ)

[5. Dickie EW, Anticevic A, Smith DE, Coalson TS, Manogaran M, Calarco N, et al. Ciftify: A framework for surface-based analysis of legacy MR acquisitions. Neuroimage. 2019;197:818–26.](http://paperpile.com/b/WcVrFf/lr0n)

[6. Burnham KP, Anderson DR. Multimodel Inference: Understanding AIC and BIC in Model Selection. Sociol Methods Res. 2004;33:261–304.](http://paperpile.com/b/WcVrFf/4DNV)
